# Intelligent Fault Diagnosis of Rolling Bearing Based on Gramian Angular Difference Field and Improved Dual Attention Residual Network

**DOI:** 10.3390/s24072156

**Published:** 2024-03-27

**Authors:** Anshi Tong, Jun Zhang, Liyang Xie

**Affiliations:** 1 School of Mechanical Engineering, Shenyang University, Shenyang 110044, China; tonganshi680@163.com; 2 School of Mechanical Engineering and Automation, Northeastern University, Shenyang 110819, China; lyxie@mail.neu.edu.cn

**Keywords:** intelligent fault diagnosis, Gramian Angular Difference Field (GADF), improved dual attention residual network (IDARN), imbalance data

## Abstract

With the rapid development of smart manufacturing, data-driven deep learning (DL) methods are widely used for bearing fault diagnosis. Aiming at the problem of model training crashes when data are imbalanced and the difficulty of traditional signal analysis methods in effectively extracting fault features, this paper proposes an intelligent fault diagnosis method of rolling bearings based on Gramian Angular Difference Field (GADF) and Improved Dual Attention Residual Network (IDARN). The original vibration signals are encoded as 2D-GADF feature images for network input; the residual structures will incorporate dual attention mechanism to enhance the integration ability of the features, while the group normalization (GN) method is introduced to overcome the bias caused by data discrepancies; and then the model is trained to complete the classification of faults. In order to verify the superiority of the proposed method, the data obtained from Case Western Reserve University (CWRU) bearing data and bearing fault experimental equipment were compared with other popular DL methods, and the proposed model performed optimally. The method eventually achieved an average identification accuracy of 99.2% and 97.9% on two different types of datasets, respectively.

## 1. Introduction

Rolling bearings are widely used in modern machinery manufacturing, which accounts for most of the market share [1]. Rolling bearings have large working loads, large differences in operating speeds and harsh working environments, so they are often prone to many kinds of failures in production [2]. Obtaining information about internal faults that may occur in bearings during operation and determining the health status of bearings through accurate and intelligent methods is a hot topic of current research [3,4].

Currently there are two types of bearing fault diagnosis methods: one is the traditional fault signal analysis method. Ding et al. proposed a gene mutation particle swarm optimisation variational modal decomposition (GMPSO-VMD) algorithm; this method can effectively deal with the problem of the fault signals of early rolling bearings being weak and difficult to extract [5]; Ye et al. used an improved empirical modal decomposition (IEMD) method applied to bearing fault diagnosis, which has good identification of weak noise and sudden impulses of bearing fault signals [6,7], and Wu et al. in order to accurately separate and extract the composite fault signal features of bearings, proposed a method combining adaptive variational modal decomposition (AVMD) and improved multivariate universe optimisation (IMUO) algorithms parameterised with maximum correlation kurtosis inverse convolution (MCKD) [8]. However, these types of methods require the mastery of advanced signal processing techniques and manual selection of sensitive features.

Another is the data-driven deep learning based method. Table 1 summarises the literature review related to the use of data-driven methods for reliability prediction in different industry applications. Zhou et al. proposed a rolling bearing fault diagnosis method based on Whale grey wolf optimization algorithm-variable modal decomposition-support vector machine (WGWOA-VMD-SVM) [9]. Zhang et al. proposed an optimised long short-term memory (LSTM) neural network fault diagnosis method for reliability analysis of gearboxes [10]; Wan et al. proposed a rolling bearing fault diagnosis method based on Spark and Improved Random Forest Algorithm [11], and Hadi et al. proposed an AutoML method to improved bearing fault classification for predictive maintenance in industrial IoT [12]. Although these types of intelligent diagnostic methods can automatically extract features, there are some unavoidable drawbacks; for example, extracting features and fault classification need to be carried out independently, and the learning efficiency is insufficient.

The emergence of convolutional neural networks (CNNs) has overcome the above drawbacks and has been favoured by many researchers. Tian et al. designed a hybrid particle swarm optimisation (HPSO) based CNN-LSTM bearing fault diagnosis model for early fault diagnosis [13]; Ye et al. proposed a TCNN for fault diagnosis of rotating machinery to address the problem of model training crash with small samples [14]. Han et al. propose a GAF-EDL method for the problem of poor model classification under noisy samples [15]. Some researchers have also used GAF in combination with CNNs. Zhou et al. proposed the bearing fault diagnosis method based on GAF and DenseNet, which achieved good results [16]; Wei et al. and Cui et al. proposed fault diagnosis models combining GAF with lightweight channel attention networks to improve classification accuracy [17,18], and Cai et al. combined GAF with deep residual networks (ResNeXt50) in order to extract more fault features [19].

Although current neural networks can achieve a decent level of classification, there are still some problems with the above methods:

1. Most methods preprocess the original signals before inputting them into the network for model training; this may filter out some important features and limit the extraction of features by the network.
2. In the traditional neural network structure, more features are extracted by stacking the layers of the network, but the network degradation phenomenon occurs and the features are not sufficiently extracted.
3. Many methods use equivalent data distributions (equal number of samples for each fault class) to train the model; whereas in real plants often the data distribution is uneven, and the model may breakdown under imbalanced data.

In order to solve the above problems, this paper proposes an intelligent diagnosis of rolling bearings based on Gramian Angular Difference Field and Improved Dual Attention Residual Network, and the main contributions are as follows:

1. The vibration signals were converted into images using the GADF method, so that the fault features were fully represented in the images; both retaining the timing information and enhancing the features to improve the network’s recognition capability.
2. The residual structure introduced into the GN was used as the network body, which solves the network degradation problem and reduces the bias caused by the difference in the distribution of training samples. Meanwhile, Dual-Attention mechanism was introduced to enhance feature integration and make full use of channels and deep information.
3. The effects of different sets of GNs and different attentional mechanisms introduced on the model were verified on the CWRU bearing dataset.
4. Small samples of imbalanced data were divided on the bearing fault experimental equipment and compared with current advanced fault diagnostic models, and the proposed method achieved the best diagnostic results in several datasets with different data distributions.

The structure of this paper is as follows. Section 2 describes the specific principles of the relevant contents. Section 3 details the proposed IDARN fault diagnosis model and the specific diagnosis process. Section 4 outlines the experimental analyses to verify the validity of the proposed model by using data collected from popular bearing datasets and faulty bearing experimental equipment. Section 5 presents the conclusions.

## 2. Related Work

This chapter provides an overview of the related methods of use. The specific implementation of GADF is presented in Section 2.1, the structure of the residual network and the specific computational process of the GN method are described in Section 2.2, and the implementation of the two attention mechanisms is explored in Section 2.3.

### 2.1. Gramian Angular Difference Field (GADF)

Gramian Angular Difference Field is a coding method that converts one-dimensional time series into two-dimensional images [20]; using it as preprocessing of vibration signals allows the network to efficiently identify vibration signals. Assuming the existence of a one-dimensional time series *X* = {*x*_1_, *x*_2_, …, *x_n_*}, the GADF coding process can be divided into the following steps, some notations used in this paper are illustrated in Table 2.

Step 1: The *X* is normalised to fall in the interval [0, 1], determined by the following equation:

(1) $${\vec{x}}_{i}=\frac{x_{i}-\min\left(X\right)}{\max\left(X\right)-\min\left(X\right)}$$

Step 2: Transform the scaled sequence data into polar coordinate system. Think of the numerical values as the angle cosines and the timestamps as the radius, representing in polar coordinates the rescaled time series *X*, determined by the following equation:

(2) $$\left\{\begin{array}{ll} \varphi_{i}=\arccos\left({\vec{x}}_{i}\right) & \left(0\leq {\vec{x}}_{i}\leq 1\right)\\ r_{i}=\frac{t_{i}}{N} & (t_{i}\in N) \end{array}\right.$$

Step 3: For the polar coordinate system φ that stores the time information, it can be encoded into the geometric structure of the matrices by calculating the trigonometric difference of each polar coordinate in the system, defined as:

(3) $$GADF=\left[\sin\left(\varphi_{i}-\varphi_{j}\right)\right]$$

(4) $$={\left(\sqrt{I-X^{2}}\right)}^{\prime }\cdot X-X^{\prime }\cdot \sqrt{I-X^{2}}$$

**Figure 1:**
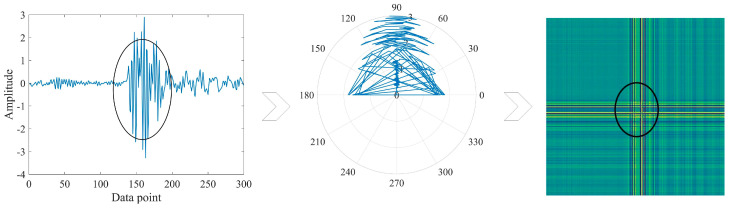
GADF coding process.

The differences in vibration signals are mainly on different time scales, which contain their own unique fault features. Figure 1 illustrates the coding process. When the one-dimensional signal has dramatic amplitude, the image corresponds to the appearance of obvious cross lines, and the larger the amplitude the more obvious the cross. GADF coding both retains the timing information and achieves the feature enhancement, which is helpful for the feature extraction of the network.

### 2.2. Network and Methodology Introduction

#### 2.2.1. Residual Neural Networks

**Figure 2:**
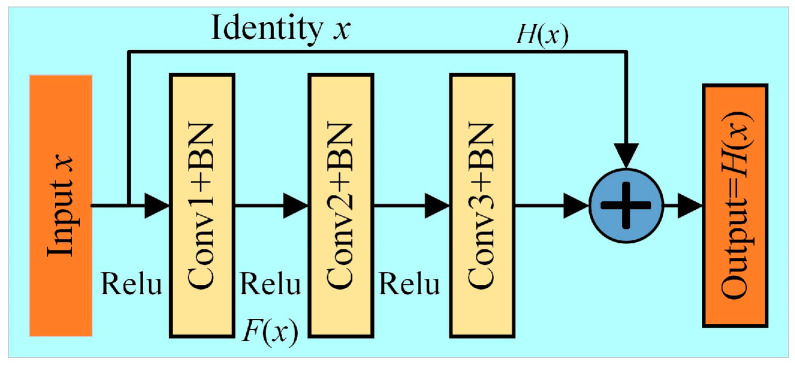
Residual structure.

In traditional convolutional neural networks, with the number of network layers, stacked models will have the problem of gradient disappearance or explosion. Residual neural networks emerged to solve this problem and have been widely used in image processing [21]; the structure is as shown in Figure 2, and determined by Equation (5).

*H*(*x*) = *F*(*x*) + *x* (5)

The residual structure can pass the features from the bottom layers of the network to the top layers directly through shortcuts, just ensuring that the input vector *x* and the residual mapping function *F*(*x*) have the same size. With the residual structure stacks, not only the depth of the convolutional layers is increased, but the network degradation problem is solved and the stability of the model is improved.

#### 2.2.2. Group Normalization Method (GN)

Residual network involves multiple layers of superposition, and the data distribution of the network can be affected by changes in parameters. Therefore, normalisation methods can be used to adjust the data distribution and effectively reduce the impact of data changes. In recent years, batch normalisation (BN) has been proposed to perform global normalisation in order to improve the training speed of neural networks. It is now widely used in fault diagnosis to improve the performance of networks.

Although BN is widely used, it is very sensitive to batch size. The calculated mean and variance are insufficient to represent the entire data distribution and may lead to poor diagnostic performance when used with small batches. Therefore, this paper introduced a new normalisation method, the GN method, to replace the BN method and eliminate the small batch effect.

In GN, it is assumed that the given input is *X* = {*x*_1_, *x*_2_, …, *x_M_*}, *X* ∈ R*_M_*_×*H*×*W*×*C*_, GN first divides the channel into *G* groups and then solves for the mean and standard deviation of each group, which can be defined as:

(6) $$\mu_{G}\left(x\right)=\frac{1}{\left(\frac{C}{G}\right)HW}\sum_{C=\frac{gC}{G}}^{\frac{\left(g+1\right)C}{G}}\sum_{h=1}^{H}\sum_{w=1}^{W}x_{mchw}$$

(7) $$\sigma_{G}\left(x\right)=\sqrt{\frac{1}{\left(\frac{C}{G}\right)HW}\sum_{c=\frac{gC}{G}}^{\frac{\left(g+1\right)C}{G}}\sum_{h=1}^{H}\sum_{w=1}^{W}{\left(x_{mchw}-u_{mg}\left(x\right)\right)}^{2}+\varepsilon }$$

Then, the function representing GN is:

(8) $$x_{mchw}=\frac{x_{mchw}-u_{G}}{\sqrt{\sigma_{G}^{2}}}$$

(9) $$y_{mchw}=\gamma x_{mchw}+\beta$$

The BN method relies on the mean and variance of the data to obtain the desired performance. When the dataset changes, the mean and variance change with it, resulting in inconsistency between the training and validation stages. However, GN relies on dividing the channels into different groups and normalise the data in each group to fit the different distribution forms of each group. Therefore, the feature matrices of the training and validation sets are accepted by each convolutional layer after GN, which guarantees a good classification ability from imbalanced similarly distributed data.

### 2.3. Attention Mechanisms

In residual networks, stacked convolutional layers can capture lots of feature data, but these data may contain excessively repetitive information, causing some performance loss. For this reason, this paper introduces two attention mechanisms to refine the feature information layer by layer from the channel dimension to the spatial dimension, which makes the network more attentive to the fault features of the image data [22,23].

#### 2.3.1. Channel Attention Mechanism

**Figure 3:**
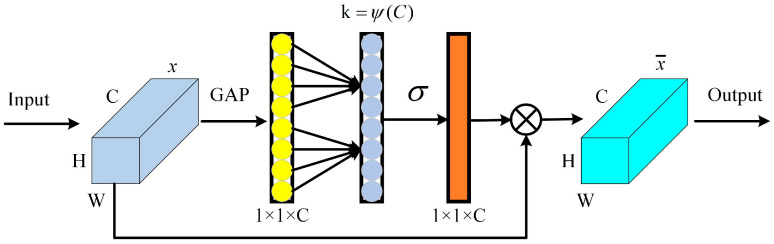
Efficient Channel Attention (ECA).

ECA is a streamlined channel attention mechanism that has been widely used in image processing. The complexity of the model is reduced by local cross-channel information interaction without dimensionality reduction; it can add weights to the features of different channels so that the network can better attention to the weights of different features, which helps in feature extraction. Figure 3 shows the ECA structure.

The following steps can be taken to realise ECA:

Firstly, the input data are subjected to Global Average Pooling (GAP) which is determined by the following equation:

(10) $$t=\frac{1}{H\times W}\sum_{i=1,\;\;j=1}^{H,W}x_{ij}$$

Secondly, feature interaction between channels is achieved by 1D convolution of Equation (11); where the size of the 1D convolution kernel *k* is determined by Equation (12):

(11) $$\omega =\sigma \left(D_{k}\left(t\right)\right)$$

(12) $$k=\Psi \left(C\right)={\left|\frac{\log_{2}\left(C\right)}{\lambda }+\frac{b}{\lambda }\right|}_{odd}$$

Finally, features with different weights are obtained after performing dot product operation of input data with channel weights. Therefore, ECA allows the integration of features in the channel dimension, making the residual network more effective in extracting fault features.

#### 2.3.2. Spatial Attention Mechanism

**Figure 4:**
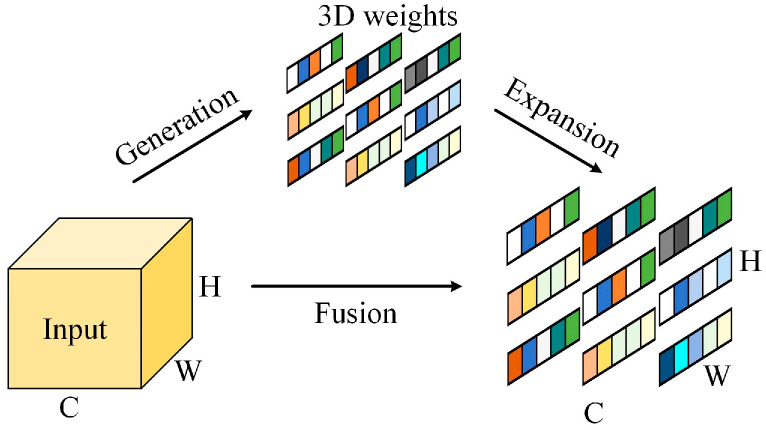
SimAM structure.

SimAM is a parameter-free spatial attention mechanism. It accurately calculates the similarity metric between features by adaptively learning and utilising the similarity information between targets so that the weights of different features can be determined; this allows more attention to be paid to different features in images and classified. The structure is shown in Figure 4.

It designs an energy function for calculating the attention weights, which can be defined as:

(13) $$e_{\alpha }=\frac{1}{S-1}\sum_{i=1}^{S-1}{\left[-1-\left(w_{\alpha }x_{i}+b_{\alpha }\right)\right]}^{2}+{\left[1-\left(w_{\alpha }\alpha +b_{\alpha }\right)\right]}^{2}+\eta w_{\alpha }^{2}$$

The importance of different features can be derived from the calculation of the energy function to achieve an enhancement effect on different classes of weak features; therefore, SimAM can focus further on the features after channel attention refinement.

## 3. Model Structure and Fault Diagnosis Process

This chapter gives an overview of the model structure of the Improved Dual Attention Residual Network and the specific process used for bearing fault diagnosis.

### 3.1. IDARN Model Structure

**Figure 5:**
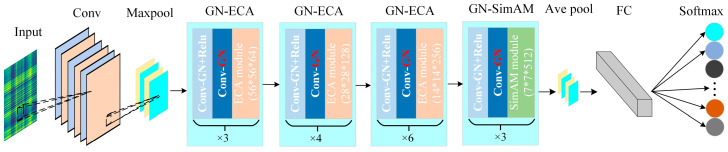
IDARN model structure.

In this section, the IDARN method for rolling bearing fault diagnosis is proposed, which is generally divided into improved residual layer, FC fully connected layer and Softmax classification layer. The specific model structure is shown in Figure 5; the parameters are shown in Table 3.

The IDARN model adopts 34-layer residual structure as the backbone, and introduced the channel and spatial attention mechanisms to extract and refine the features layer by layer from the channel to the spatial dimension; meanwhile, the GN method is also introduced in the residual layers to adapt to the different distribution forms of each set of data. In this way, the network has excellent ability to identify and classify different weak fault features, and effectively reduces the computational errors brought by different data distributions.

### 3.2. Fault Diagnosis Process

**Figure 6:**
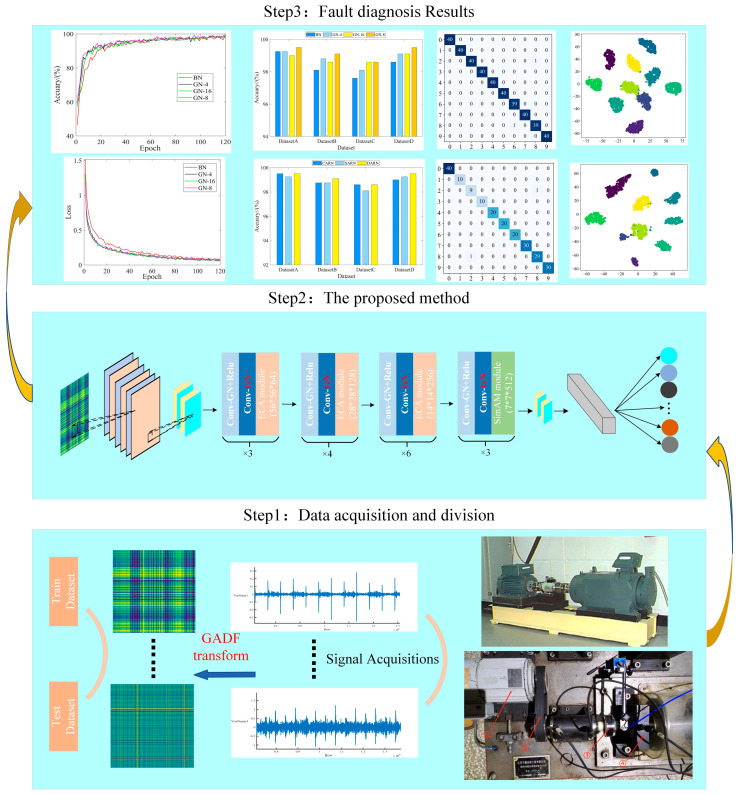
Fault diagnosis process.

Figure 6 demonstrates the general process of rolling bearing diagnosis based on GADF and Improved Dual Attention Residual Network. It can be divided into the following steps:

Step 1: The original signals collected from the bearing failure experimental equipment were converted into two-dimensional images after GADF encoding, and the corresponding data sets were divided.

Step 2: Set the network parameters and input the divided datasets into improved dual attention residual network for training.

Step 3: The influence of the different number of GN groups on the model was verified in several datasets, then divided and compared with other attentional mechanisms.

Step 4: Comparison of existing popular CNN models on the small-sample unbalanced datasets was performed to validate the superiority of the proposed method.

## 4. Experimental Analysis

In this chapter, data obtained from CWRU bearing data and the bearing failure experimental equipment were used to evaluate the effectiveness of the diagnostic methods. All network models were trained in Pytorch framework using Python 3.8 programming with Intel Core i5-7300 CPU@2.5 GHz and GTX1050(4G) under Windows 10.

### 4.1. Case 1: CWRU Bearing Dataset Analysis

#### 4.1.1. Data Processing

**Figure 7:**
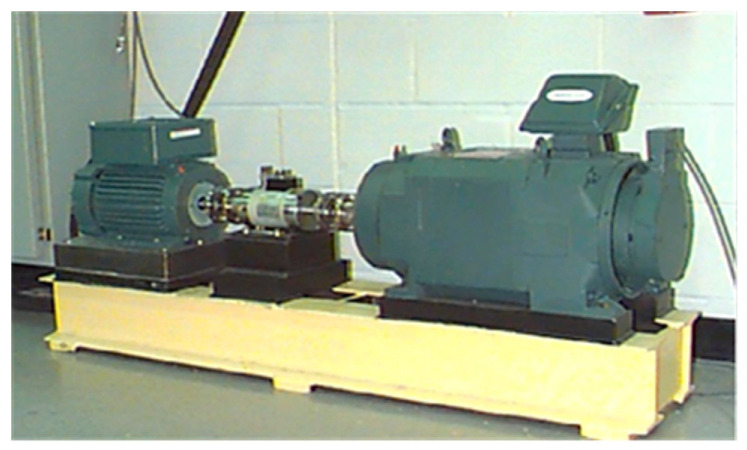
Experimental equipment of CWRU [24].

The experimental setup is shown in Figure 7. Taking the drive-end bearing SKF-6205 as example, the experimenter set different failure diameters of damage to the outer ring (@3, @6, @12), inner ring, and rolling body of the bearing respectively. Acceleration sensors were installed on the drive end bearing housing to collect fault signals with a sampling frequency of 12 kHz. In this paper, a load of 0.75 kw was selected, and the corresponding rotational speed of the motor was 1772 r/min.

Three different damage diameters of the inner ring, outer ring (@6), and rolling body types were selected as the failure data samples, and one health state data sample was also selected, which was divided into ten categories, and the different failure types were recorded as “IF”, “OF (@6)”, “RE”, and “NO”. It is worth noting that DatasetB/C/D were imbalanced datasets, simulating the missing data in real working conditions; DatasetA can be regarded as a balanced dataset in the ideal state. To avoid the effects of chance, the 400 data points in each category were randomly assigned in a ratio of 9:1 for training and validation. For the imbalanced data, 100/200/300 GADF data were randomly selected from the measured signal segments; again, these were randomly assigned in a 9:1 ratio, and the validity of the proposed model classification was verified using the datasets in Table 4.

#### 4.1.2. Data Preprocessing and Network Training Settings

The data points were collected using a sliding window for translational sliding on the one-dimensional data while generating GADF-encoded images. To ensure that each image contains sampling points for one week of bearing rotation, the size of the sliding window was calculated using the following equation.

(14) $$Q=\frac{60\times F_{q}}{R}$$

From the above equation, the bearing samples approximately 400 data points per revolution; therefore, the sliding window size was set to 400 to sample with the smallest sliding window.

In order to ensure data utilisation and signal integrity, data enhancement was used to expand the data. The data enhancement method used in this paper was to overlap the samples of the original one-dimensional sequences by taking a sliding window with a step size of 200 for overlap sampling. Since each input sequence was obtained in a single fault state, the enhanced samples have the same fault labels as the original sequence.

**Figure 8:**
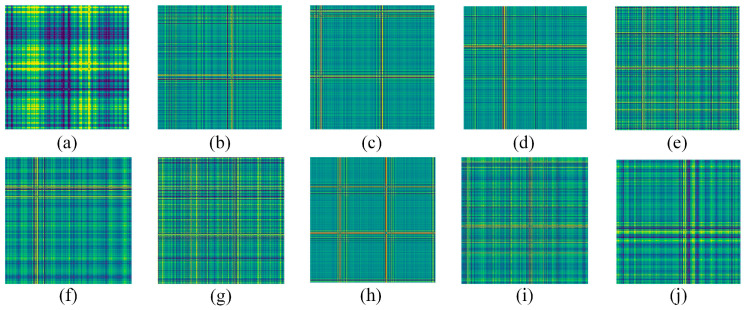
Fault coded images. (**a**) NO; (**b**) IF-0.007in; (**c**) IF-0.014in; (**d**) IF-0.021in; (**e**) RE-0.007in; (**f**) RE-0.014in; (**g**) RE-0.021in; (**h**) OF-0.007in; (**i**) OF-0.014in; (**j**) OF-0.021in.

**Figure 9:**
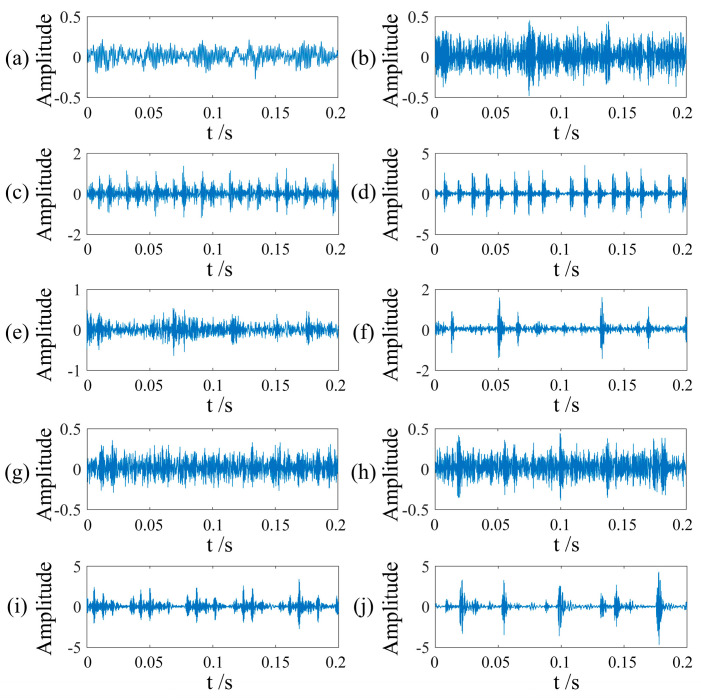
Vibration images of faults. (**a**) NO; (**b**) RE-0.007in; (**c**) IF-0.007in; (**d**) OR-0.007in; (**e**) RE-0.014in; (**f**) IR-0.014in; (**g**) OF-0.014in; (**h**) RE-0.021in; (**i**) IF-0.021in; (**j**) OF-0.021in.

Finally, considering the effect of hardware devices, the sliding window was panned once to generate a 300 × 300 GADF coded image. Figure 8 shows the fault images of different categories, each of which has obvious features that can be used for fault classification. Figure 9 shows the vibration images of different bearings under different faults. The network training parameters were shown in Table 5.

#### 4.1.3. Analysis of Different Methods

**Figure 10:**
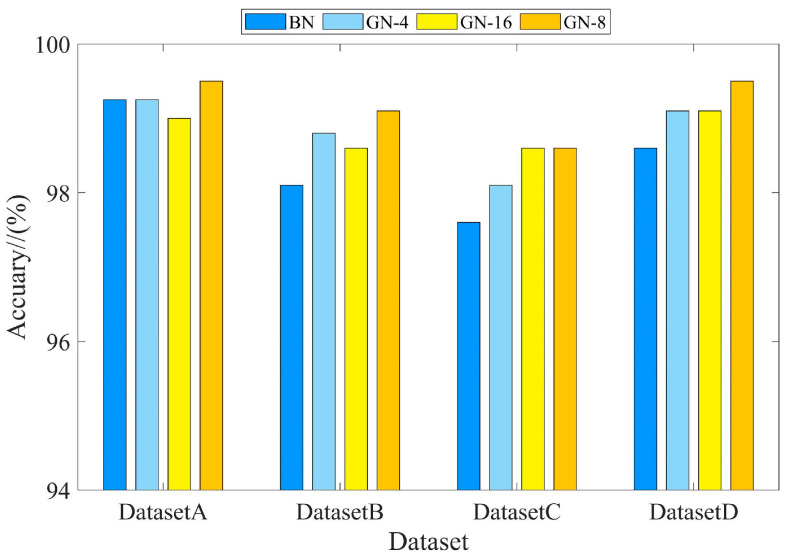
Comparison of different normalisation methods.

**Figure 11:**
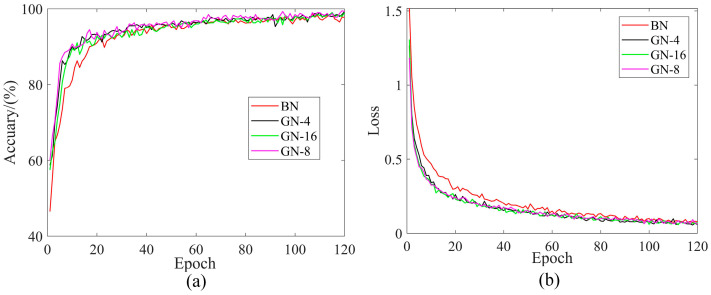
Training performance curves of BN and GN method comparison. (**a**) Accuracy (DatasetA); (**b**) Loss (DatasetA).

The BN and GN methods were introduced for comparison under four datasets to highlight the superiority of the GN method. The number of groups in GN has an impact on the performance of the model, so different numbers of groups for the GN method were considered in this experiment. Each training iteration was 120 rounds and the training parameters are shown in Table 5. Figure 10 and Table 6 show the results of comparing the two methods with four datasets. For further analysis, DatasetA was taken as an example, and the corresponding training curves are shown in Figure 11.

As can be seen from the Figure 10, the introduced GN method was better than the BN method and achieved the optimal classification accuracy under the four datasets. As shown in Figure 11b, the training loss of the GN method was lower than BN. More importantly, the GN method can overcome the bias caused by data discrepancy, while the BN method cannot, and thus the GN had to perform better under the four datasets.

**Figure 12:**
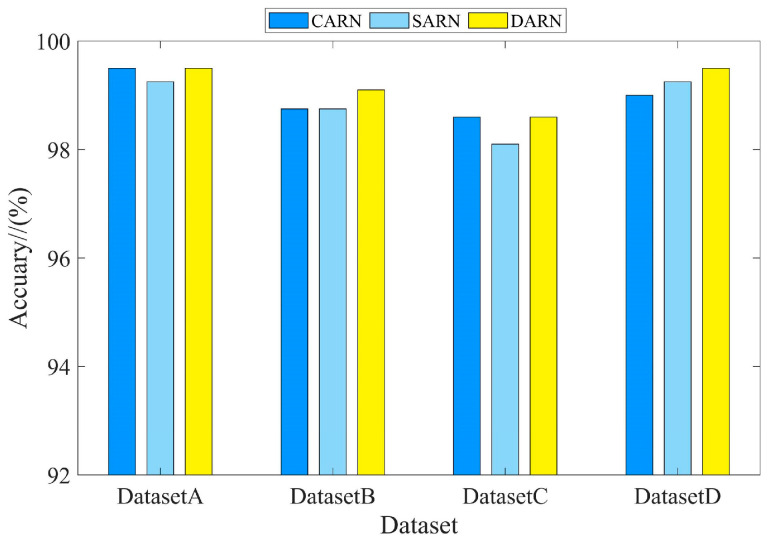
Comparison of different methods of attention.

In order to verify the superiority of the dual attention mechanism for feature extraction, the channel (CARN) or spatial (SARN) attention methods were compared under the same GN method. Each dataset was trained for 120 rounds, and the comparison results are shown in Figure 12 and Table 7. From these results, it can be concluded that the dual attention mechanism was able to focus on more features and achieve the highest classification accuracy under the four datasets.

#### 4.1.4. Comparison with Related Models (CWRU Failure Data)

**Figure 13:**
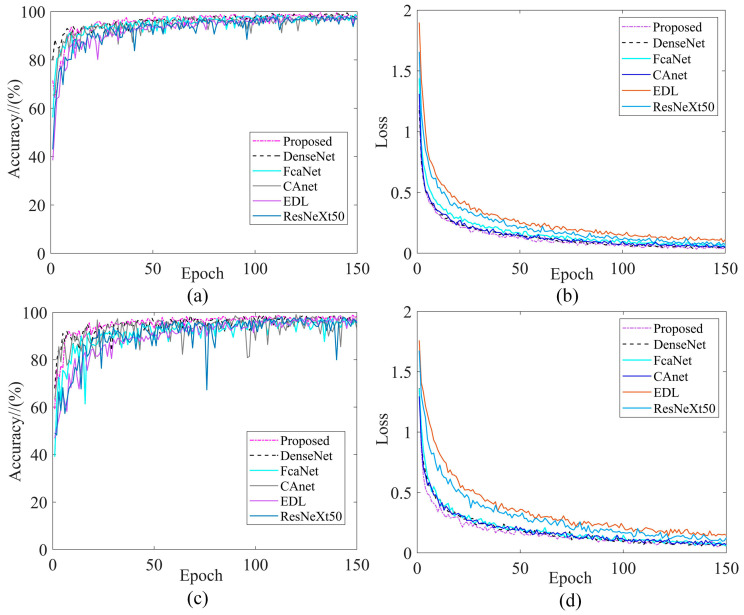
Training performance curves. (**a**) Accuracy/DatasetA; (**b**) Loss/DatasetA; (**c**) Accuracy/DatasetB; (**d**) Loss/DatasetB.

In recent years, many researchers have also used GAF in combination with other DL models for fault diagnosis. To demonstrate the superiority of combining GADF with IDARN, we compared the methods of references [15,16,17,18,19] with the proposed method, using the same GADF processing, input to different models for training. To ensure each model converged sufficiently, each model was iterated 150 times, taking DatasetA and DatasetB (one balanced and one imbalanced data) as examples, Figure 13 illustrates the training performance curves for the different methods.

As can be seen from Figure 13a, all models on DatasetA were relatively stable when trained and gradually converge. In the first 20 rounds of training, the training accuracy of proposed model was not as good as that of DenseNet; whereas after 20 rounds of training, the two models were almost equal and the stability of fluctuation was better than that of the other four models. From Figure 13b, it can be seen that the training loss of the present model was lowest at 10–100 rounds of training, and at 100–150 rounds it was similar to CAnet and DenseNet, but in general the proposed model can achieve the lowest training loss.

As can be seen from Figure 13c,d, all models were trained with significantly higher fluctuations for imbalanced data than for balanced data, and the training loss was slightly higher relative to the balanced dataset. This can also reflect the impact of the amount of data on model training in deep learning, but for the proposed method, a good training result can still be achieved on imbalanced datasets, with a steady convergence.

Evaluation indicators are standards for judging the performance of diagnostic algorithms and are important in data analysis. In the field of deep learning fault diagnosis, Accuracy (*A_c_*), Precision (*P_r_*), Recall (*R_e_*), and *F*1 score are all standards for judging the performance of the model, and the expressions are as in Equations (15)–(18). In order to analyse the advantages and shortcomings of each model, Table 8 shows the classification accuracy on the four datasets, Table 9, Table 10, Table 11 and Table 12 shows the different indicators for the different models in DatasetA/B/C/D. For a more comprehensive analysis, we also refer to the training time.

(15) $$A_{c}=\frac{TP+TN}{TP+FP+TN+FN}$$

(16) $$P_{r}=\frac{TP}{TP+FP}$$

(17) $$R_{e}=\frac{TP}{TP+FN}$$

(18) $$F1=\frac{2\times P_{r}\times R_{e}}{P_{r}+R_{e}}$$

where: *TP* and *TN* are the number of correct predictions in *i* categories; *FP* and *FN* are the number of incorrect predictions in *i* categories.

From Table 9, on the balanced dataset, it can be seen that the proposed method was slightly lower than DenseNet in the three indicators, which was due to the fact that DenseNet uses a deeper network (121 layers, while the proposed method is 34 layers) to extract features resulting in a better classification of a particular label than the proposed model, but its training time was not as good as that of the proposed model. The other comparative methods were lower than the proposed model in all the indicators.

From Table 10, Table 11 and Table 12, on the imbalanced datasets, it can be seen that the proposed method has slightly lower *R_e_* on DatasetB and *P_r_* on DatasetC than DenseNet, and outperforms the other methods on DatasetD for all indicators. Combining all the indicators, the proposed method has clear advantages in terms of imbalanced data.

#### 4.1.5. Analysis of Results Compared with Different Methods

In recent years, many researchers have focused on fault diagnosis of balanced datasets. In order to further prove the superiority of the IDARN method, this section was compared with some commonly used methods. The comparison results were shown in Table 13. The proposed method achieves the highest classification accuracy of 99.5% with balanced datasets. Compared with references [25,26,27,28,29], the IDARN method identifies more fault types and substantially improves the classification accuracy.

#### 4.1.6. Visualisation and Analysis

**Figure 14:**
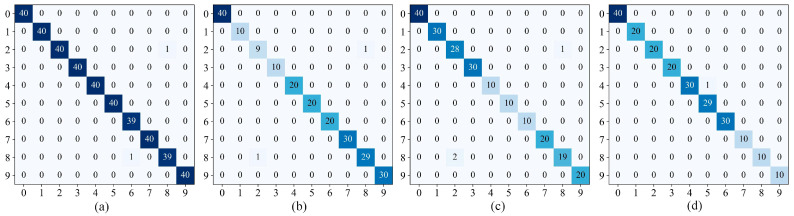
Confusion matrices. (**a**) DatasetA; (**b**) DatasetB; (**c**) DatasetC; (**d**) DatasetD.

Figure 14 showed the classification confusion matrix of the IDARN model under the four datasets. The *y* label in the confusion matrix represents the predicted label, the *x* label represents the true label, and the numbers on the diagonal represent the overlap between the true label and the predicted label. Using the validation set of different datasets as an example, a specific categorisation of each fault can be derived. It can be seen that in DatasetA label 6 and label 8 had the lowest accuracy; in DatasetB/C label 2 and label 8 had the lowest accuracy; and in DatasetD only label 5 had the lowest accuracy. Some labels had misclassification because there may be similar features between samples, but overall IDARN had good classification performance.

**Figure 15:**
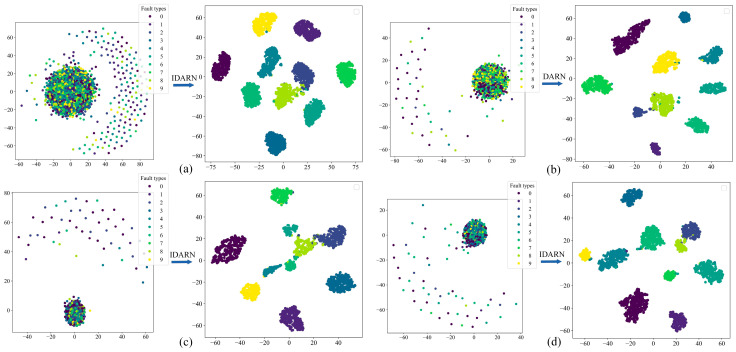
t-SNE. (**a**) DatasetA; (**b**) DatasetB; (**c**) DatasetC; (**d**) DatasetD.

In order to demonstrate the classification effect of this model more intuitively, several datasets were visualised and analysed using the t-SNE method, as shown in Figure 15. In the left half of each subfigure is the original feature distribution of the training set for each dataset, and the right half is the classification result of the fully connected layer after the training set of each dataset has been trained by IDARN. As can be seen from the figures, the original cluttered features distribution of the dataset becomes uniformly clustered through this training method, and there was almost no substantial overlap between each category. As such, the method had good classification effect on the balanced/unbalanced data of rolling bearings.

### 4.2. Case 2: Data Analysis of Variable Speed Bearing Fault Experimental Equipment

#### 4.2.1. Data Collection and Segmentation

**Figure 16:**
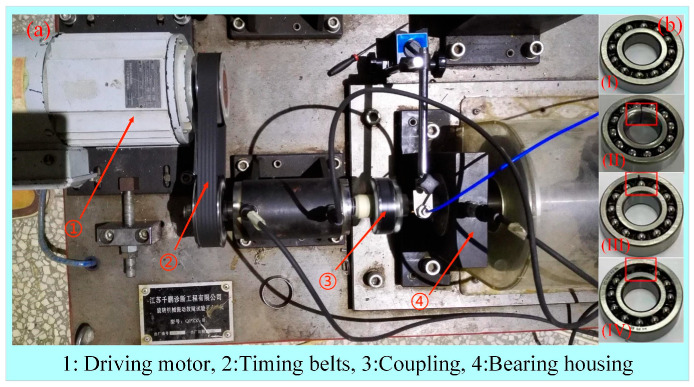
Bearing fault experimental equipment. (**a**) Experimental equipment (QPZZ-II); (**b**) Different states of the bearings: (I) Normal; (II) Failure of the inner ring; (III) Failure of the outer ring; (IV) Roller body failure.

The experimental data were obtained from the bearing fault experimental equipment (QPZZ-II), as shown in Figure 16a. According to the practical application, an acceleration sensor was placed on the bearing housing to collect the vibration data of the bearing. In the experiment, the sampling time of the sensor was set to 15 s each time, and the sampling rate was 20480 S/s. Depending on the designed fault scenes, these data were labelled as “Normal (NO)”, “Inner Ring Fault (IF)”, “Outer Ring Fault (OF)”, and “Rolling Element Failure (RE)”, and the different bearing states were shown in Figure 16b.

Type of bearings in the experiment: HRB-1205ATN self-aligning ball bearings, the number of rolling elements: 12 (single row). Bearing size: outer diameter is 52 mm, inner diameter is 25 mm, inner ring diameter is 33.3 mm, width is 15 mm.

Generation and shape of bearing defects: (1) Failure of the inner ring: processing by wire-cut method until the inner ring breaks (width about 1 mm) (Figure 16(b-II)). (2) Failure of the outer ring: a groove with a width of 1 mm and a depth of about 1 mm is machined inside the outer ring of the bearing by wire-cutting method (Figure 16(b-III)). (3) Roller body failure: randomly removed two adjacent balls (Figure 16(b-IV)).

Bearing operating conditions: Temperature of 20 °C and speed range of 75–1500 rpm (500/1000/1500 rpm in the experiment, respectively). No load, lubricant soaked, then 1/2 grease applied.

**Figure 17:**
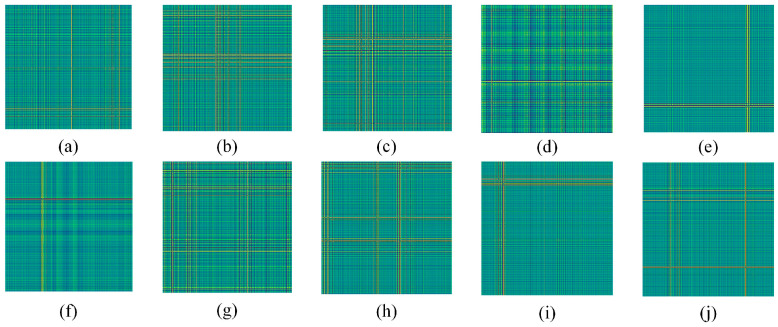
Fault coded images. (**a**) NO; (**b**) IF-500; (**c**) IF-1000; (**d**) IF-1500; (**e**) OF-500; (**f**) OF-1000; (**g**) OF-1500; (**h**) RE-500; (**i**) RE-1000; (**j**) RE-1500.

According to Equation (14), the smallest sliding window was taken for sampling. Different faults with different rotational speeds were used to divide the corresponding datasets; considering the imbalanced fault samples and fewer samples in practice, the imbalanced datasets with small samples were divided. For the imbalanced data, to avoid the effects of chance, 100/150/200 GADF data were randomly selected from the measured signal segments; again, these were randomly assigned in a 9:1 ratio as shown in Table 14. The corresponding GADF coded images generated were shown in Figure 17.

#### 4.2.2. Comparative Experimental Analysis

**Figure 18:**
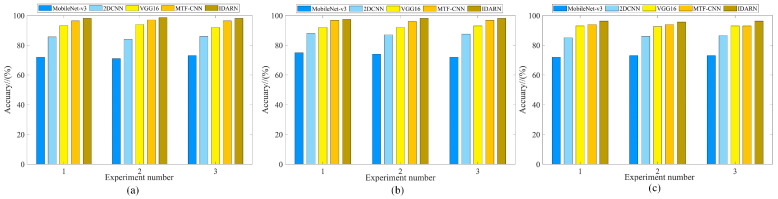
Comparison results. (**a**) Dataset1; (**b**) Dataset2; (**c**) Dataset3.

In this section, IDARN used a grouping form of G = 8. Several popular fault diagnosis methods were compared under three datasets to highlight the superiority of the present method. Liang et al. proposed a 2DCNN approach applied to fault diagnosis of rotating machinery [30]; Wu et al. proposed a VGG16 approach for fault diagnosis of high-voltage DC transmission lines [31]; Wang et al. proposed the MTF-CNN for fault diagnosis of rolling bearings [32], and Gu et al. used MobileNet-v3 network for automatic fault detection of variable speed bearings [33]. Each method was trained for 200 rounds each time, and all the experiments were trained 3 times to avoid the effect of randomness. The experimental results are shown in Figure 18 and Table 15. It can be seen that the recognition accuracies of MobileNet-v3 and 2DCNN under the three datasets were 72.2% and 85.5%, respectively, which was an obvious change that can reflect the superiority of the method.

**Figure 19:**
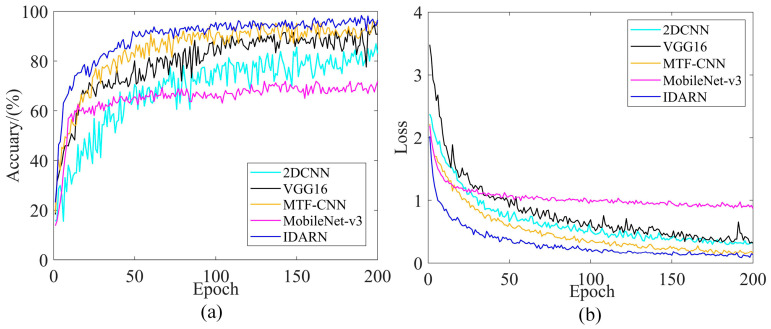
Training performance curves for popular model comparisons. (**a**) Accuracy (Dataset1); (**b**) Loss (Dataset1).

Taking Dataset1 as an example, the corresponding training performance curves were shown in Figure 19. It can be seen that the two training performances of IDARN were better than other advanced CNN models. It is worth noting that the Mobilenet-v3 model cannot achieve good classification results after 200 iterations, and more iterations were needed; the rest of the models all converge stably.

The different indicators on the three datasets are shown in Table 16, Table 17 and Table 18. As can be seen from the table, although several other models had shorter training times, other indicators were not as good as the proposed model. This is due to the fact that their inherent shallow structure does not allow for better extraction of image features. Therefore, IDARN can identify more fault features and achieve the best classification results with a limited number of iterations.

#### 4.2.3. Comparison with Related Models (QPZZ-II Bearing Failure Data)

**Figure 20:**
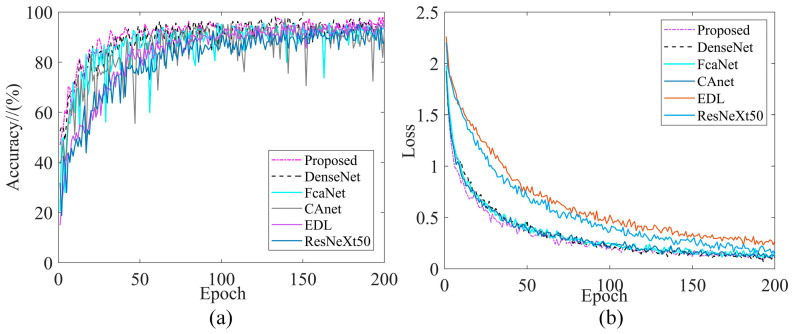
Training performance curves for related model comparison. (**a**) Accuracy (Dataset2); (**b**) Loss (Dataset2).

In order to further compare the advantages and disadvantages of different models, take Dataset2 as an example. Comparing the literature [15,16,17,18,19] under the same training conditions, the training performance curves are shown in Figure 20. Table 19, Table 20 and Table 21 shows several indicators corresponding to each model.

From Figure 20a, it can be seen that the accuracy of DenseNet was higher than the proposed model in the initial stage of training, and the proposed model was higher than the other models when the training reached 40–50 rounds, and after 60 rounds of training the two models, DenseNet and IDARN were almost equal. It is worth noting that the other models occasionally jumped around during the overall training process and were not as stable as the proposed model.

From Figure 20b, it can be seen that the EDL model had the highest loss, while the loss of IDARN in the first 50 rounds of training was significantly lower than the other models, and the training loss of IDARN was slightly lower than that of DenseNet at iterations 50–200 rounds. This was due to the deeper structure of DenseNet, but overall, the average loss was essentially equal to that of the proposed model.

As can be seen from Table 19, Table 20 and Table 21, DenseNet and IDARN achieved the highest accuracy on Dataset2, while IDARN had the shortest training time. Comparing all the indicators on the three datasets, the model proposed in this paper still outperforms the other models, therefore IDARN has good robustness under small sample imbalanced data.

#### 4.2.4. Visualisation and Analysis of Results

**Figure 21:**
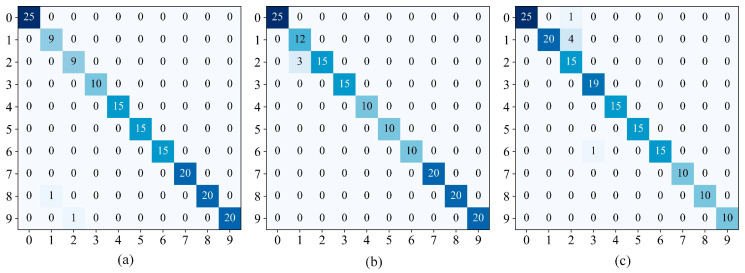
Confusion matrices. (**a**) Dataset1; (**b**) Dataset2; (**c**) Dataset3.

**Figure 22:**
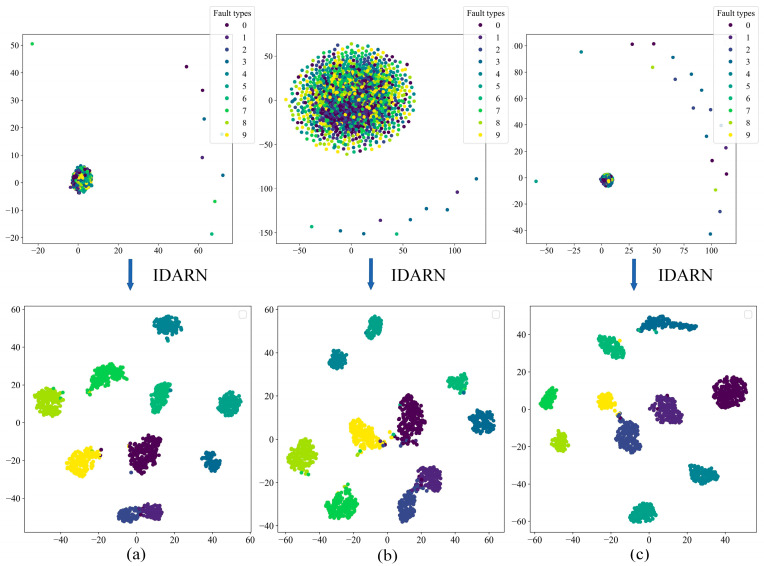
t-SNE. (**a**) Dataset1; (**b**) Dataset2; (**c**) Dataset3.

Figure 21 shows the confusion matrices of the proposed method under the three datasets. It can be seen that label 1 and label 2 have the lowest accuracy under the three datasets and the rest of the labels achieve full classification. Figure 22 shows the results obtained by the proposed method after t-SNE clustering under the three datasets; it can be seen that there was no confounding between the different categories and the faults were classified well. Therefore, IDARN can be used for fault classification of small sample imbalanced data.

## 5. Conclusions

To address the problems of deep network models degradation and the collapse of model training when the data distribution is imbalanced; this paper proposes an intelligent diagnosis of rolling bearings based on Gramian Angular Difference Field and Improved Dual Attention Residual Network, and mainly draws the following conclusions:

1. Using GADF to convert one-dimensional signals into two-dimensional images preserves the correlation of the time series and enhances the shock signature; which is more beneficial for the network’s identification of fault features.
2. The residual structure introduced into the GN is used as the body of the network, which solves the network degradation problem and reduces the bias caused by the difference in the distribution of the training samples; meanwhile, the dual-attention mechanism introduced can enhance the integration ability of the features.
3. The effects of different normalisation methods and different attention mechanisms on the model were validated on the CWRU bearing dataset. An average recognition accuracy of 99.2% was achieved with the four datasets divided.
4. An average identification accuracy of 97.9% was obtained on small samples of imbalanced data divided on the bearing fault experimental equipment.

Although the GADF-IDARN method can obtain good fault diagnosis performance, this method is trained from scratch and therefore the present method requires a longer training cycle compared to other shallow neural network methods. In further work, transfer learning methods can be utilised for fault diagnosis tasks to reduce training time. In addition, the GADF-IDARN methodology should also be utilised to perform on a wider range of datasets.

## Figures and Tables

**Table 1:** Summary of literature of data-driven methods models.

Ref.	Application	Model	Brief Description	Shortage
[9]	Rolling bearing	WGWOA-VMD-SVM	The proposed method can extract more features.	Difficult to train samples on a large scale.
[10]	Gearboxes	VMD-LSTM	Solved EMD modal overlap problem.	Longer calculation time.
[11]	Rolling bearing	Spark-IRF	Solve the problems of slower diagnosis speed and repeated voting of traditional RF algorithm.	Overfitting on some noisy classification or regression problems.
[12]	Industrial IoT	AutoML	To address the need for bearings to effectively manage predictive maintenance applications.	Not effective when not learning.
[13]	Rolling bearing	HPSO-CNN-LSTM	For early fault diagnosis of bearings.	Excessive convolutional layers degrade the network.
[14]	Rotating machinery	TCNN	Excellent diagnostic results for small sample data.	Need to pre-train the model.
[15]	Rolling bearing	GAF-EDL	For diagnosing data under noise	Not applicable to regression problems.
[16,17,18,19]	Rolling bearing	GAF + CNN	To lighten the model and extract more features.	Unstable during training with imbalanced data.

**Table 2:** Commonly used notations.

Notations	Descriptions
*X*	One-dimensional time series
φ	Polar coordinates of the angle cosine
*t_i_*	Timestamp
*N*	Constant factor
*I*	Row vector
*x*	Input data
*H*(*x*)	Residual mapping function
*F*(*x*)	Constant mapping function
*M*	The size of the batch
*H*	The height of the feature matrices
*W*	The width of the feature matrices
*C*	The number of channels
*G*	Groups of the channels
$\mu_{G}\left(x\right)$	Mean deviation for each group
$\sigma_{G}\left(x\right)$	Standard deviation for each group
*γ*	Scale parameter
*β*	Conversion parameter
*x_ij_*	Feature matrices under the channels *i* and *j*
*t*	Global aggregated features
ω	Weight assigned to each channel
σ	Sigmoid function
*D*	1D convolution operation
${\left|\cdot \right|}_{odd}$	The closest odd number to the variable
*b* and λ	Constants
*S;*	The number of neurons on each channel
*xi*	Different neuron
α	The neuron for the input feature of each channel
$w_{\alpha }$ and $b_{\alpha }$	The weights and bias values
η	Constant taken as 1 × 10^−4^
*Q*	The number of sampling points
*F_q_*	The sampling frequency
*R*	The rotational speed of the bearing

**Table 3:** IDARN model parameters.

Disposition	Output Dimension	Layer Design
Input	224 × 224 × 3	——
Conv	112 × 112 × 64	7 × 7 × 64, *s* = 2
Max pool	56 × 56 × 64	3 × 3, *s* = 2
GN-ECA-1	56 × 56 × 64	$\left[\begin{array}{c} 3\times 3\times 64\\ 3\times 3\times 64 \end{array}\right]\times 3$
GN-ECA-2	28 × 28 × 128	$\left[\begin{array}{c} 3\times 3\times 128\\ 3\times 3\times 128 \end{array}\right]\times 4$
GN-ECA-3	14 × 14 × 256	$\left[\begin{array}{c} 3\times 3\times 256\\ 3\times 3\times 256 \end{array}\right]\times 6$
GN-SimAM	7 × 7 × 512	$\left[\begin{array}{c} 3\times 3\times 512\\ 3\times 3\times 512 \end{array}\right]\times 3$
Avg pool	1 × 1 × 512	7 × 7, *s* = 1
FC	1 × 1 × 1000	——
Softmax	10	——

**Table 4:** Division of the datasets.

Condition	FD/(in)	Label	DatasetA	DatasetB	DatasetC	DatasetD	Train/Val
NO	0	0	400	400	400	400	
	0.007	1	400	100	300	200	
IF	0.014	2	400	100	300	200	
	0.021	3	400	100	300	200	
	0.007	4	400	200	100	300	9/1
RE	0.014	5	400	200	100	300	
	0.021	6	400	200	100	300	
	0.007	7	400	300	200	100	
OF (@6)	0.014	8	400	300	200	100	
	0.021	9	400	300	200	100	

**Table 5:** Network training parameters.

Pre-Processing Method	Batch Size	Loss Function	Optimizer	Learn Rate
Random Horizon Filp				
Random Resize Crop	8	Crossentropy Loss	Adam	0.0001
Normalize				

**Table 6:** Accuracy of different normalisation methods.

Dataset/Method	BN/(%)	GN-4/(%)	GN-8/(%)	GN-16/(%)
DatasetA	99.3	99.3	99.5	99
DatasetB	98.1	98.8	99.1	98.6
DatasetC	97.5	98.1	98.6	98.6
DatasetD	98.6	99.1	99.5	99.1

**Table 7:** Accuracy of different attention methods.

Dataset/Method	CARN/(%)	SARN/(%)	DARN/(%)
DatasetA	99.5	99.3	99.5
DatasetB	98.6	98.8	99.1
DatasetC	98.6	98.1	98.6
DatasetD	99.1	99.2	99.5

**Table 8:** Comparative accuracy in validation set.

Method/Dataset	DatasetA/(%)	DatasetB/(%)	DatasetC/(%)	DatasetD/(%)
GADF-EDL [15]	99	98.6	98.2	98.2
GADF-DenseNet [16]	99.5	98.2	98.6	99.1
GADF-FcaNet [17]	99.3	98.2	98.2	98.6
GADF-CAnet [18]	99	98.6	97.7	98.2
GADF-ResNeXt50 [19]	98.5	97.7	98.2	98.6
GADF-IDARN	99.5	99.1	98.6	99.5

**Table 9:** Comparative indicators in DatasetA.

Method	*P_r_* (Avg)	*R_e_* (Avg)	*F*1	Train Time/Epoch
GADF-EDL	0.9894	0.9902	0.9898	106 s
GADF-DenseNet	0.9952	0.9950	0.9951	145 s
GADF-FcaNet	0.9926	0.9925	0.9925	108 s
GADF-CAnet	0.9904	0.9900	0.9901	111 s
GADF-ResNeXt50	0.9856	0.9850	0.9853	266 s
GADF-IDARN	0.9950	0.9950	0.9950	96 s

**Table 10:** Comparative indicators in DatasetB.

Method	*P_r_* (Avg)	*R_e_* (Avg)	*F*1	Train Time/Epoch
GADF-EDL	0.9887	0.9817	0.9852	62 s
GADF-DenseNet	0.9714	0.9867	0.9790	94 s
GADF-FcaNet	0.9714	0.9800	0.9757	63 s
GADF-CAnet	0.9853	0.9824	0.9838	83 s
GADF-ResNeXt50	0.9733	0.9733	0.9733	146 s
GADF-IDARN	0.9920	0.9833	0.9876	54 s

**Table 11:** Comparative indicators in DatasetC.

Method	*P_r_* (Avg)	*R_e_* (Avg)	*F*1	Train Time/Epoch
GADF-EDL	0.9853	0.9750	0.9801	62 s
GADF-DenseNet	0.9885	0.9817	0.9851	94 s
GADF-FcaNet	0.9809	0.9850	0.9829	63 s
GADF-CAnet	0.9747	0.9703	0.9725	83 s
GADF-ResNeXt50	0.9818	0.9800	0.9809	146 s
GADF-IDARN	0.9871	0.9883	0.9877	54 s

**Table 12:** Comparative indicators in DatasetD.

Method	*P_r_* (Avg)	*R_e_* (Avg)	*F*1	Train Time/Epoch
GADF-EDL	0.9847	0.9809	0.9828	62 s
GADF-DenseNet	0.9909	0.9933	0.9921	94 s
GADF-FcaNet	0.9887	0.9803	0.9845	63 s
GADF-CAnet	0.9853	0.9824	0.9838	83 s
GADF-ResNeXt50	0.9885	0.9824	0.9854	146 s
GADF-IDARN	0.9968	0.9967	0.9967	54 s

**Table 13:** Comparative results.

Method	Classification Category	Accuracy (%)
1D-CNN [25]	6	93.2
CNNEPDNN [26]	10	97.85
LSTM-1DCNN [27]	10	98.46
MTF-ResNet [28]	10	98.52
ResNet-LSTM [29]	10	98.95
IDARN	10	99.5

**Table 14:** Data division.

Condition	Rpm	Label	Dataset1	Dataset2	Dataset3	Train/Val
NO	1000	0	250	250	250	
	500	1	100	150	200	
IF	1000	2	100	150	200	
	1500	3	100	150	200	
	500	4	150	100	150	9/1
OF	1000	5	150	100	150	
	1500	6	150	100	150	
	500	7	200	200	100	
RE	1000	8	200	200	100	
	1500	9	200	200	100	

**Table 15:** Accuracy of different methods.

Dataset/Method	Mobilenet-v3/%	2DCNN/%	VGG16/%	MTF-CNN/%	IDARN/%
Dataset1	71.9	85.6	94.6	97	98.8
Dataset2	74	87.2	92.8	96.4	98.1
Dataset3	70.7	83.8	93.4	94	96.9

**Table 16:** Comparative indicators of popular CNN models in Dataset1.

Method	*P_r_* (Avg)	*R_e_* (Avg)	*F*1	Train Time/Epoch
Mobilenet-v3	0.7293	0.7175	0.7233	17 s
2DCNN	0.8493	0.8300	0.8395	35 s
VGG16	0.9414	0.9093	0.9251	26 s
MTF-CNN	0.9684	0.9726	0.9704	57 s
IDARN	0.9904	0.9800	0.9852	37 s

**Table 17:** Comparative indicators of popular CNN models in Dataset2.

Method	*P_r_* (Avg)	*R_e_* (Avg)	*F*1	Train Time/Epoch
Mobilenet-v3	0.7526	0.7367	0.7446	17 s
2DCNN	0.8633	0.8468	0.8550	35 s
VGG16	0.9307	0.9169	0.9237	26 s
MTF-CNN	0.9749	0.9603	0.9675	57 s
IDARN	0.9833	0.9800	0.9816	37 s

**Table 18:** Comparative indicators of popular CNN models in Dataset3.

Method	*P_r_* (Avg)	*R_e_* (Avg)	*F*1	Train Time/Epoch
Mobilenet-v3	0.7149	0.7056	0.7102	17 s
2DCNN	0.8267	0.8347	0.8307	35 s
VGG16	0.9252	0.9336	0.9294	26 s
MTF-CNN	0.9537	0.9430	0.9483	57 s
IDARN	0.9733	0.9700	0.9716	37 s

**Table 19:** Comparative Indicators of related models in Dataset1.

Method	Accuracy (%)	*P_r_* (Avg)	*R_e_* (Avg)	*F*1	Train Time/Epoch
GADF-EDL	97.7	0.9756	0.9700	0.9728	45 s
GADF-DenseNet	98.1	0.9816	0.9749	0.9782	53 s
GADF-FcaNet	97	0.9747	0.9667	0.9707	44 s
GADF-CAnet	96.9	0.9696	0.9567	0.9631	48 s
GADF-ResNeXt50	95.6	0.9633	0.9566	0.9599	107 s
GADF-IDARN	98.8	0.9904	0.9800	0.9852	37 s

**Table 20:** Comparative Indicators of related models in Dataset2.

Method	Accuracy (%)	*P_r_* (Avg)	*R_e_* (Avg)	*F*1	Train Time/Epoch
GADF-EDL	96.3	0.9605	0.9493	0.9549	45 s
GADF-DenseNet	98.1	0.9826	0.9733	0.9779	53 s
GADF-FcaNet	95.6	0.9449	0.9383	0.9416	44 s
GADF-CAnet	97.5	0.9707	0.9660	0.9683	48 s
GADF-ResNeXt50	95	0.9507	0.9220	0.9361	107 s
GADF-IDARN	98.1	0.9833	0.9800	0.9816	37 s

**Table 21:** Comparative Indicators of related models in Dataset3.

Method	Accuracy (%)	*P_r_* (Avg)	*R_e_* (Avg)	*F*1	Train Time/Epoch
GADF-EDL	93.1	0.9409	0.9403	0.9406	45 s
GADF-DenseNet	95.6	0.9665	0.9650	0.9657	53 s
GADF-FcaNet	94.4	0.9538	0.9550	0.9544	44 s
GADF-CAnet	95.6	0.9652	0.9650	0.9651	48 s
GADF-ResNeXt50	92.5	0.9252	0.9393	0.9322	107 s
GADF-IDARN	96.9	0.9733	0.9700	0.9716	37 s

## Data Availability

Case Western Reserve University Bearing Data. https://engineering.case.edu/bearingdatacenter (accessed on 25 September 2023).
